# The End of *Personification*: The Mereological Fallacy in Science Communication on Brain Organoids

**DOI:** 10.1080/15265161.2023.2278564

**Published:** 2024-01-18

**Authors:** Sietske A. L. van Till, Eline M. Bunnik

**Affiliations:** Erasmus MC, University Medical Centre Rotterdam

In the last two decades, stem cell-based brain organoids have been developed to study disease mechanisms in various neurological, psychiatric, and developmental disorders. Simultaneously, there have been discussions in the bioethical literature on the ‘moral status’ of these models, which center mainly on their presumed potential to develop some form of consciousness (De Jongh et al. 2022). In her Target Article, Jennifer Blumenthal-Barby (2024) argues that in discussions on the use of new technological entities, including chimeras, uploaded minds, artificial intelligence, and also brain organoids, bioethicists should stop using the concept of personhood, because it is unhelpful in the attempt to determine how these entities should be treated in practice. Instead, bioethicists should use other concepts, such as ‘interests’ or ‘sentience’, to determine whether new technological entities can be wronged, and ‘respect’, to determine what they are owed. In her article, Blumenthal-Barby mentions brain organoids only in passing. In this commentary, we apply and extend her argument to brain organoids and other neuronal cell models, including two-dimensional neuronal networks. We demonstrate that also in discussions on how brain organoids and other neuronal cell models ought to be treated, the use of the concept of personhood is a ‘problematic shortcut’. In addition, we argue that *implicit personifications* of brain organoids and other neuronal cell models, e.g., by comparing or equating these models with human beings, can likewise feed into unjustified assumptions about their moral status. We expose these implicit personifications as instances of the so-called ‘mereological fallacy’—i.e., the tendency to attribute human-like properties to the brain,—and show that they are omni-present in science communication on brain organoids. We warn the research community against the effects of this fallacy, and offer recommendations for science communication on brain organoid research.

Mereology is the study of relationships between ‘wholes’ and ‘parts’, and, thus, a mereological fallacy is a logically invalid form of reasoning that mistakes parts for wholes (Bennett and Hacker 2003). Bennett and Hacker explain the lack of validity in this argument as follows: “psychological predicates which apply only to human beings or (other animals) as wholes cannot intelligibly be ascribed to their parts, such as the brain” (Bennett and Hacker 2003, 73). Examples of the mereological fallacy in the context of neuroscience include ‘the brain remembers X’, ‘the brain loves X’, or ‘the brain is addicted to X’. Mereological fallacy can be widely observed in science communication on brain organoids. We illustrate this with two examples.

First, Kagan et al. (2022) recently integrated *in vitro* neuronal networks into a closed-loop feedback computer system simulating the well-known computer game ‘pong’. Following the publication of their scientific paper titled “In vitro neurons learn and exhibit sentience when embodied in a simulated game-world,” numerous news articles were published in (prominent) newspapers, including the BBC, the Guardian, and the New Scientist, using headlines such as: “A dish of brain cells figured out how to play pong in 5 minutes” (Starr 2022). In this later article Kagan stated that “We’re trying to create a dose response curve with ethanol—basically get them ‘drunk’ and see if they play the game more poorly, just as when people drink.” The publication contains pictures of human beings playing pong.

The publication by Kagan et al. is criticized by other neuroscientists because of the “unsupported use of terms and concepts [such as “sentience,” “goal-directed behavior,” “embodiment,” and “intelligence”] that misrepresent the findings of this study” (Balci et al. 2023). The use of such terms to describe neuronal cell models is an example of the mereological fallacy at play. For in this publication, neuronal cell models are ‘personified’ by describing them as intentional subjects with human-like properties. Neuronal cell models are attributed certain mental states we typically preserve for describing humans, such as being ‘drunk’ or being ‘intelligent’. Also, these models are portrayed as subjects that can act intentionally, by ‘playing’ games, or ‘figuring out’ how to perform a specific task. The fact that neuronal cells act or react in measurable ways, does not mean that they intend to perform a task and understand how to do it. Likewise, it cannot be assumed that neuronal cell models experience joy such as we, as human beings, do when we play games. Let alone that neuronal cell models ‘look’ like humans playing pong, as is suggested by the pictures of humans playing pong.

Second, in a scientific paper on the presence of electroactivity in maturing brain organoids, Trujillo et al. (2019) stated that “Cortical organoid network dynamics mimic those of premature neonates after 28 weeks of maturation.” In both the bioethical literature and public media, this paper is often referred to when it is highlighted that there is a need to anticipate the development of potentially conscious brain organoids in the future (De Jongh et al. 2022).

In the bioethical literature, brain organoids are often considered a morally distinct subgroup of organoids and ascribed special ‘moral status’, because of their presumed capacity to develop consciousness. Consciousness is sometimes treated as *on par* with electroactivity in the brain. For non-neuroscientists, above research results are easily interpretated as brain organoids having functional capacities similar to those of the brains of newborns. However, this would be an oversimplified conclusion. The scientific community agrees that, at this point, brain organoids do not show any signs of consciousness, and will probably not do so in the future (Hyun et al. 2022). Activity that can be observed on the cellular level does not imply that mental or cognitive phenomena occur (Bennett and Hacker 2003). Ascribing (the possibility of) consciousness to brain organoids disregards the significance of the physical body of the organism as a whole in establishing human-like experiences and mental or cognitive phenomena. Doing so is therefore a mereological fallacy. Some neuroscientists and bioethicists openly reject the equation of neuronal cells in a dish with conscious entities. A proposal has been made to avoid the term ‘mini-brains’ in discussions on brain organoids (Hyun et al. 2022).

The mereological fallacy, as illustrated by the above two examples, can be unhelpful and potentially harmful in the following three ways. First, (mis)representations of brain organoids can fuel ethical concerns among the general public, and negatively shape public attitudes toward brain organoids. They may lead people to mistake brain organoids for human-like subjects. This would indeed be a problematic shortcut in assessing the moral ‘considerability’ of brain organoids and the corresponding moral duties we hold toward them, e.g., when we generate, store, and use them in research settings. Undue ‘personification’ of brain organoids might lead publics to demand constraints in research use of brain organoids for specific applications (e.g., for the development of human-animal chimeras or so-called connectoids), and it might even provoke public resistance to brain organoid research generally.

Second, the mereological fallacy may fuel what is called *neurohype* (Lilienfeld et al. 2017). As previously shown, brain organoid research is often portrayed in exaggerated manners in the public media. Exaggerated (mis)representations of brain organoid research, such as ungrounded provocative scientific claims and the focus on unrealistic applications of brain organoids can contribute to *neurohype*. Personification of brain organoids should be avoided, as *neurohype* poses a threat to the scientifical credibility of neuroscientists (Lilienfeld et al. 2017).

Finally, in accordance with Blumenthal-Barby, we argue that in bioethical discussions on how brain organoids should be treated in research settings, relevant normative questions are missed because of the mereological fallacy. Elsewhere, we have shown how the hyperfocus of bioethicists on the presumed potential of brain organoids to develop consciousness leads to other, more urgent, ethical questions being overlooked (Van Till et al. 2023). These include questions on how brain organoid research can yield social value, what responsibilities researchers hold toward donors of pluripotent stem cells, and on how brain organoids can be treated *with respect* in research settings.

Given these adverse impacts of the mereological fallacy on brain organoid research, we advocate for the depersonification of brain organoids in science communication. We endorse Lilienfelds’ recommendation to “be skeptical of the attribution of human-like attributes, such as thinking and feeling, to the brain” (Lilienfeld et al. 2017, 257). We offer the following recommendations. It is important for scientists, journalists, and science communicators to establish a uniform terminology in neuroscientific communication to prevent ungrounded, oversimplified, and misleading claims in the public and scientific discourse. Terms such as ‘consciousness’ and ‘mini-brains’ are already rejected in the context of brain organoids. We additionally propose that in describing morphological and functional features of brain organoids, researchers should refrain from using terms that are generally used to refer to human-like abilities, characteristics, and experiences: neuronal cells *cannot* talk to each other, play, or learn. Bioethical discussions should align better with the scientific state of the art in organoid research. It is the responsibility of bioethicists to focus on *realistic* applications of brain organoid technology, and prioritize ethical issues that are relevant not to science fiction but to neuronal cell models that are currently being used in research settings.

## References

[CIT0001] Balci, F., S. Ben Hamed, T. Boraud, S. Bouret, T. Brochier, C. Brun, J. Y. Cohen, E. Coutureau, M. Deffains, V. Doyère, et al. 2023. A response to claims of emergent intelligence and sentience in a dish. *Neuron* 111 (5):604–5. doi:10.1016/j.neuron.2023.02.009. 36863319

[CIT0002] Bennett, M. R., and P. M. S. Hacker. 2003. *Philosophical Foundations of Neuroscience*. Oxford: Blackwell.

[CIT0003] Blumenthal-Barby, J. 2024. The end of personhood. *The American Journal of Bioethics* 24 (1):3–12. doi:10.1080/15265161.2022.2160515. 36635972

[CIT0004] De Jongh, D., E. K. Massey, VANGUARD Consortium, and E. M. Bunnik. 2022. Organoids: A systematic review of ethical issues. *Stem Cell Research & Therapy* 13 (1):337. doi:10.1186/s13287-022-02950-9. 35870991 PMC9308907

[CIT0005] Hyun, I., J. C. Scharf-Deering, S. Sullivan, J. D. Aach, P. Arlotta, M. L. Baum, G. M. Church, A. Goldenberg, H. T. Greely, P. Khoshakhlagh, et al. 2022. How collaboration between bioethicists and neuroscientists can advance research. *Nature Neuroscience* 25 (11):1399–401. doi:10.1038/s41593-022-01187-2. 36258039

[CIT0006] Kagan, B. J., A. C. Kitchen, N. T. Tran, F. Habibollahi, M. Khajehnejad, B. J. Parker, A. Bhat, B. Rollo, A. Razi, and K. J. Friston. 2022. In vitro neurons learn and exhibit sentience when embodied in a simulated game-world. *Neuron* 110 (23):3952–69.e8. doi:10.1016/j.neuron.2022.09.001. 36228614 PMC9747182

[CIT0007] Lilienfeld, S. O., E. Aslinger, J. Marshall, and S. Satel. 2017. Neurohype: A field guide to exaggerated brain-based claims. In *The Routledge Handbook of Neuroethics*, ed. L. S. M. Johnson and K. S. Rommelfanger, 241–261. New York: Routledge/Taylor & Francis Group.

[CIT0008] Starr, M. 2022. WATCH: A dish of brain cells figured out how to play pong in 5 minutes. *Science Alert*. Accessed October 23, 2023. https://www.sciencealert.com/watch-a-dish-of-brain-cells-figured-out-how-to-play-pong-in-5-minutes

[CIT0009] Van Till, S. A. L., M. V. Maksimova, G. J. M. W. van Thiel, and E. M. Bunnik. 2023. An assessment of the moral value of neuronal cell models and brain organoids. *Molecular Psychology: Brain, Behavior, and Society* 2:15. doi:10.12688/molpsychol.17557.1.

[CIT0010] Trujillo, C. A., R. Gao, P. D. Negraes, J. Gu, J. Buchanan, S. Preissl, A. Wang, W. Wu, G. G. Haddad, I. A. Chaim, et al. 2019. Complex oscillatory waves emerging from cortical organoids model early human brain network development. *Cell Stem Cell* 25 (4):558–69.e7. doi:10.1016/j.stem.2019.08.002. 31474560 PMC6778040

